# Supplementary material for: Genomic-Thermodynamic Phase Synchronization: Maxwell’s Demon-like Regulation of Cell Fate Transition
Source: Int J Mol Sci. 2025 May 20;26(10):4911. doi: 10.3390/ijms26104911 (PMC12112187; doi:10.3390/ijms26104911)
Supplement: Supplementary file 1 [file ijms-26-04911-s001.zip › ijms-3529882-supplementary.pdf]

Supplemental File:

Our information-thermodynamics analysis (ITA) demonstrates that the CP functions as a Maxwell's demon in genome regulation. Two additional supporting results, labeled **I** and **II**, are provided in this Supplemental File.

I) <u>Independent Replicate Result</u> (Replicate 2) for HRG-Stimulated MCF-7 Cells

Based on our **information-thermodynamics analysis** (ITA), we present results from Replicate 2 of the MCF-7 gene expression dataset under HRG stimulation [Saeki et al., 2009], available in the Gene Expression Omnibus (GEO) under accession ID **GSE13009**. The figure numbers correspond to those used in the main text. The results, <u>consistent with those from</u> **<u>Replicate 1</u>** (main text), reveal CP–PES phase synchronization accompanied by the activation of the Maxwell's demon function, as detailed below:

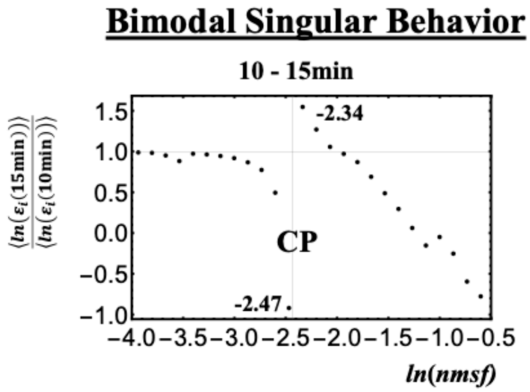

Figure S1. Identification of the critical point (CP) region. The CP region exhibits bimodal singular behavior at 10-15 min. The peak range spans from -2.47 to -2.34 in *ln*(*nrmsf*). The CP region is defined as -2.5 < *ln*(*nrmsf*) < -2.3, encompassing 3,598 genes (see the **Figure 2** caption in the main text for details).

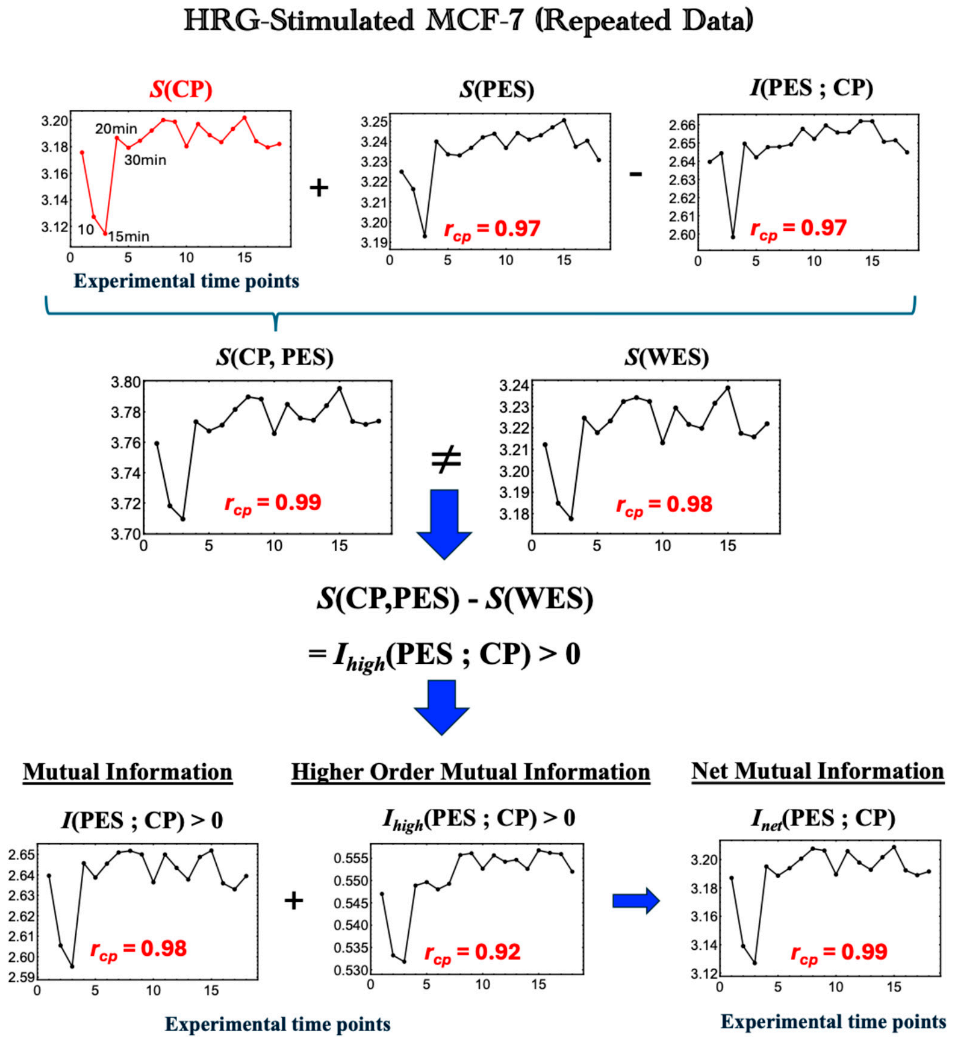

Figure S2. Thermodynamic phase synchronization and higher-Order mutual information in HRG-stimulated MCF-7 cells (Replicate 2). Temproal Peason correlation with *S*(CP) (r_{cp}) is shown in red text. The procedure follows that described in Figure 5 in the main text.

Figure S2 reveals:

1. **CP-PES synchronization is also evident in Replicate 2** for HRG-stimulated MCF-7 cells, as demonstrated by an almost perfect temporal Pearson correlation between the entropy of the critical point, ***S*(CP)**, and the net mutual information, **I_{net}(CP;PES).**
2. A positive higher-order mutual information **I_{high}(CP;PES) > 0** indicates cooperative thermodynamic behavior between the critical point (CP) and the peripheral expression system (PES). The net mutual information, **I_{net}(CP;PES)** exhibits a pulse-like pattern, <u>coinciding with the timing of a critical transition in the genome engine mechanism</u>

[Tsuchiya et al., 2020, 2022, 2023a]. The following figure visualizes this pulse-like behavior in $\boldsymbol{I_{net}}$**(CP;PES),** revealing a three-step process involving a Maxwell's demon-like action [Parrondo et al., 2015] that rewrites chromatin as dynamic memory.

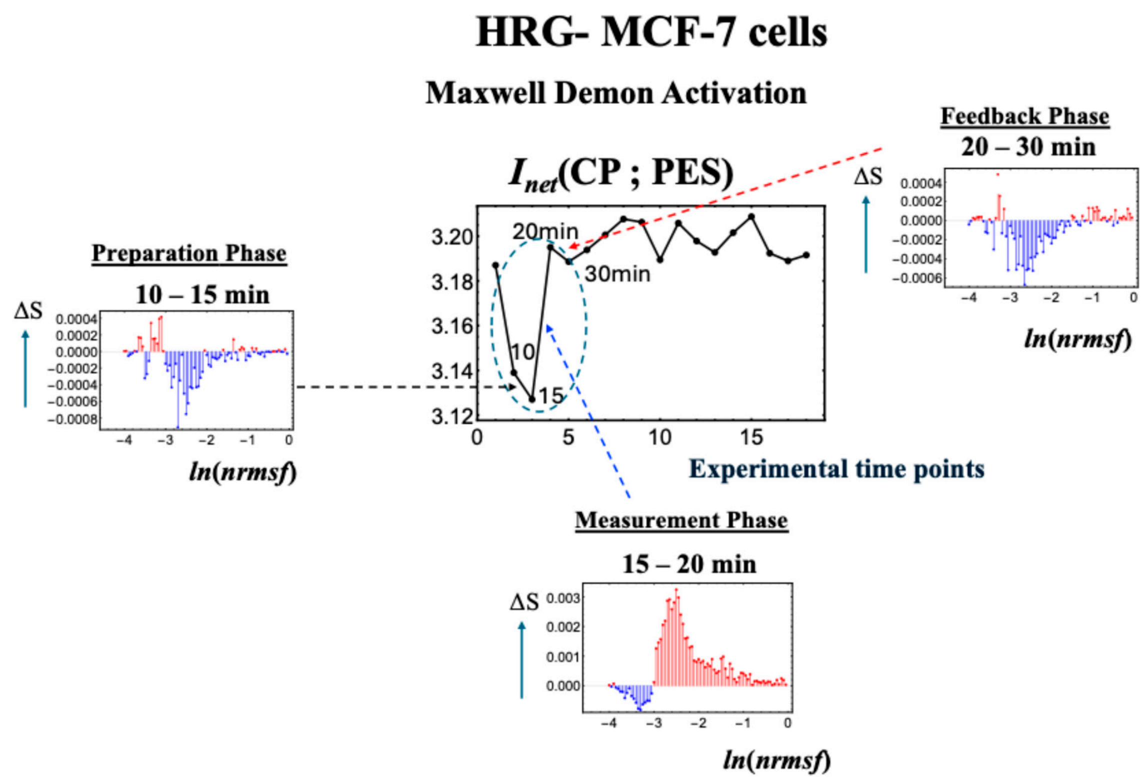

Figure S3. Maxwell's demon phases in HRG-stimulated MCF-7 Cells (Replicate 2). The net mutual information, I_{net}(CP; PES), under HRG stimulation reveals all three operational phases of a Maxwell's demon: **preparation** (10-15 min), **measurement** (15–20 min), and **feedback associated with the critical transition** (20-30 min), accompanied by coherent chromatin remodeling dynamics. (see **Figure 9** caption in the main text for details). This result supports the findings from Replicate 1 in our study.

II) DMSO-Stimulated HL-60 Human Leukemia Cells:

Using GEO ID: **GSE14500** (N = 12,625 mRNAs; details in [Huang et al., 2005]) across 13 time points: $t_1 = 0$, $t_2 = 2$ h, 4, 8, 12, 18, 24, 48, 72, 96, 120, 144, $t_{13} = 168$ h, ITA reveals pulse-like behavior in $\boldsymbol{I_{net}}$**(CP; PES),** exhibiting a three-step process involving a Maxwell's demon-like action that rewrites chromatin as dynamic memory. This further demonstrates that pluse-

like net mutual information reflects the CP's function as a Maxwell’s demon in genome regulation.

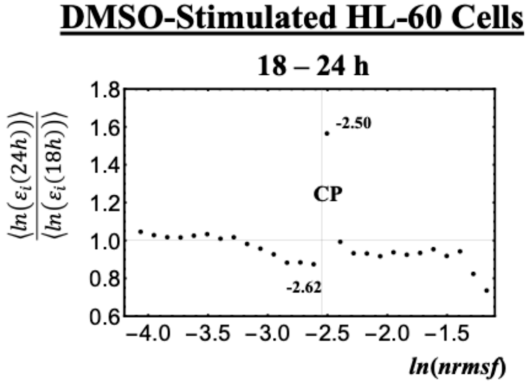

Figure S4. Identification of the critical point (CP) region. The CP region exhibits bimodal singular behavior at 18 -24 h. The peak range spans from -2.62 to -2.50 in *ln*(*nrmsf*). The CP region is defined as -2.65 < *ln*(*nrmsf*) < -2.45, encompassing 1,719 genes (see the **Figure 2** caption in the main text for details).

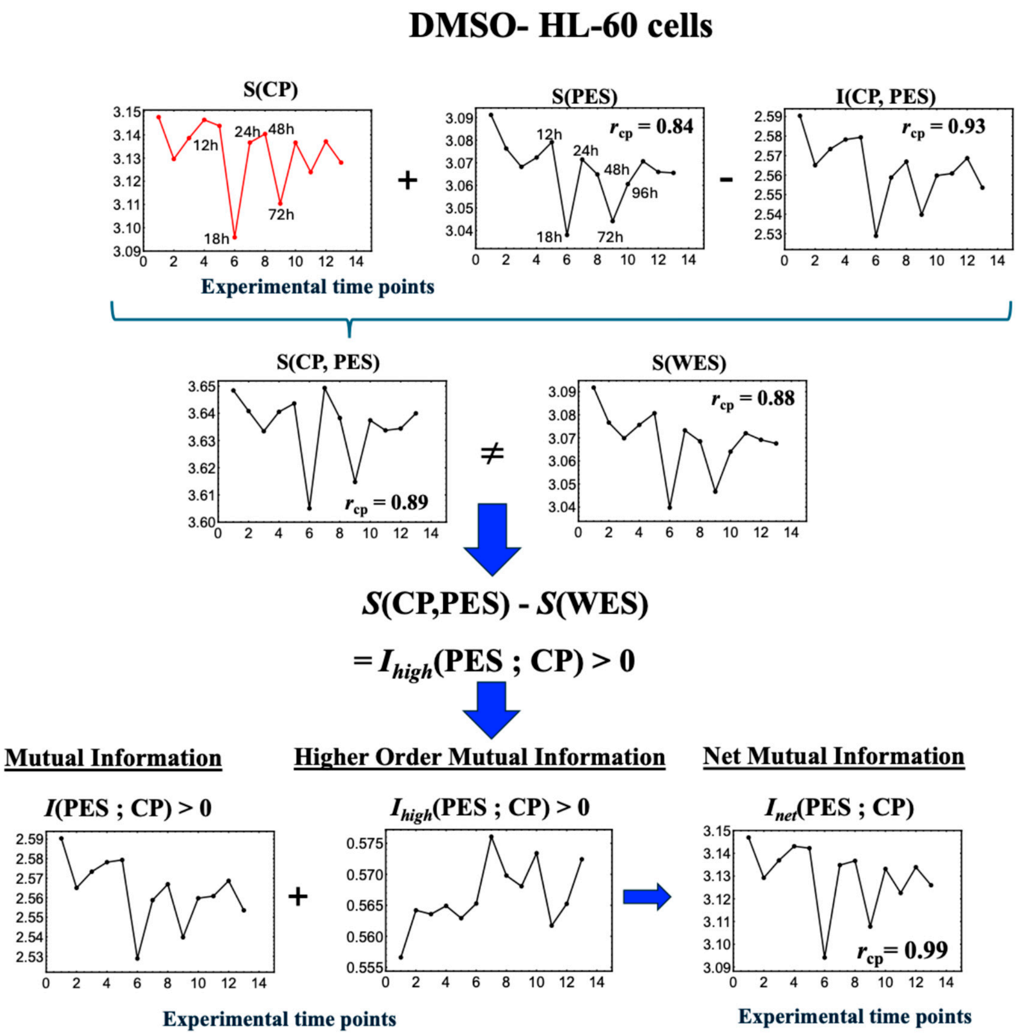

Figure S5. Thermodynamic phase synchronization and higher-order mutual information in DMSO-stimulated HL-60 cells. Thermodynamic phase synchronization and positive higher-order mutual information are also evident in DMSO-stimulated HL-60 cells, indicating cooperative behavior between the critical point (CP) and the peripheral expression system (PES).

Figure S6 visualizes the pulse-like behavior in $\boldsymbol{I_{net}}$**(CP; PES),** revealing a three-step process involving a Maxwell's demon-like action that rewrites chromatin as dynamic memory. The net mutual information, $\boldsymbol{I_{net}}$**(CP; PES),** exhibits a pulse-like pattern at 12-18 h (**preparation**), 18-24 h (**measurement**), and 48-72 h (**feedback**), accompanied by **coherent chromatin remodeling**. The critical transition corresponding to feedback occurs at 48 h.

Note: The genome engine mechanism exhibits global expression changes (genome avalanche) at 12-18 h and 18-24 h, corresponding to the pulse-like changes in I_{net}(CP; PES) observed in

Figure S6. Genome engine switching occurs at 18 h, indicating a critical transition at this time point (see Figs. 6 and 14 in [Tsuchiya et al., 2020]). ITA combined with chromatin state analysis (**Figure 7** in the main text) reveals that a time delay exists between genome engine switching and the onset of the critical transition.

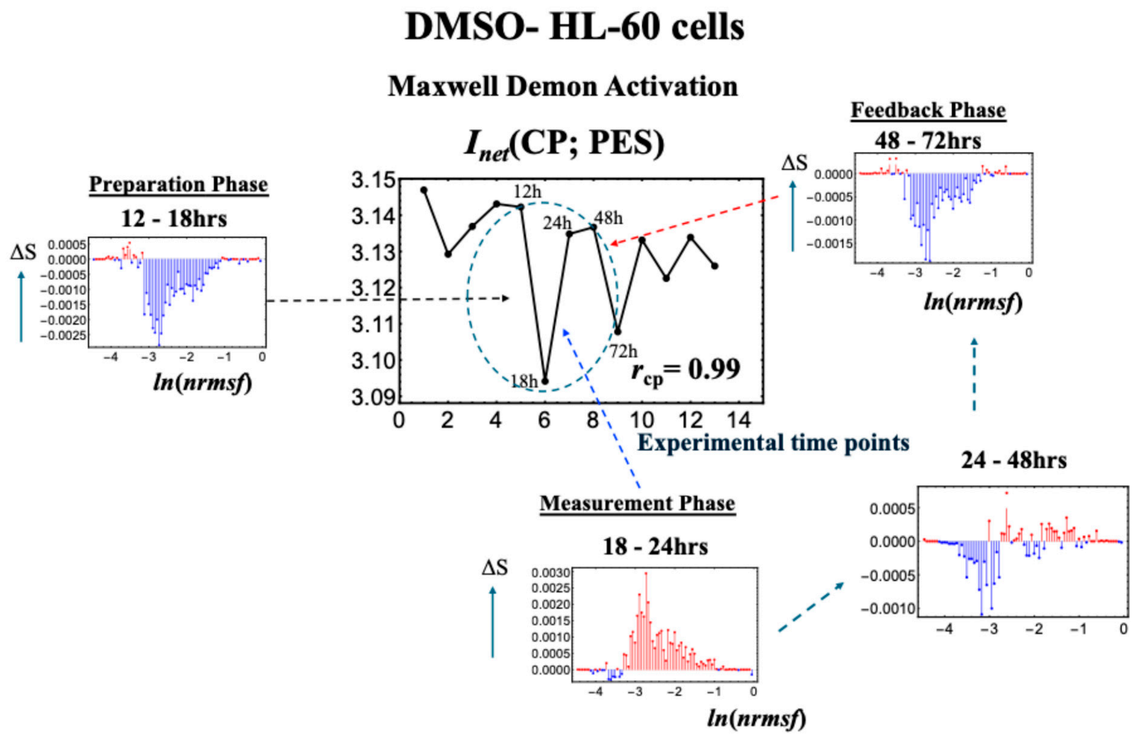

Figure S6. Maxwell demon function as rewritable chromain memory in DMSO stimulated HL-60 Cells. The net mutual information **I_{net}(CP;PES)** reveals a three-phase process **- preparation (12-18 h)**, **measurement (18-24 h)**, and **feedback (48-72 h) -** characteristic of a dynamic Maxwell's demon-like mechanism. This process reflects the function of rewritable chromatin memory during the critical transition induced by DMSO stimulation.

References:

Huang, S.; Eichler, G.; Bar-Yam, Y.; Ingber, D. E. Cell fates as high-dimensional attractor states of a complex gene regulatory network. Phys. Rev. Lett. **2005**, 94, 128701.

Parrondo, J. M. R.; Horowitz, J. M.; Sagawa, T. Thermodynamics of information. Nat. Phys. **2015**, 11, 131–139. https://doi.org/10.1038/nphys3230

Saeki, Y.; Endo, T.; Ide, K.; Nagashima, T.; Yumoto, N.; Toyoda, T.; Suzuki, H.; Hayashizaki, Y.; Sakaki, Y.; Okada-Hatakeyama, M.; et al. Ligand-specific sequential regulation of transcription factors for differentiation of MCF-7 cells. BMC Genomics **2009**, 10, 545. https://doi.org/10.1186/1471-2164-10-545

Tsuchiya, M.; Giuliani, A.; Yoshikawa, K. Cell-fate determination from embryo to cancer development: Genomic mechanism elucidated. Int. J. Mol. Sci. **2020**, 21, 4581–4617. https://doi.org/10.3390/ijms21134581

Tsuchiya, M.; Giuliani, A.; Zimatore, G.; Erenpreisa, J.; Yoshikawa, K. A unified genomic mechanism of cell-fate change. Results Probl. Cell Differ. **2022**, 70, 35–69. https://doi.org/10.1007/978-3-031-06573-6_2

Tsuchiya, M.; Brazhnik, P.; Bizzarri, M.; Giuliani, A. Synchronization between attractors: Genomic mechanism of cell-fate change. Int. J. Mol. Sci. **2023a**, 24, 11603. https://doi.org/10.3390/ijms241411603
